# Prevalence of dyslipidemia, atherogenic and cardiovascular risk in overweight and obese adolescents

**DOI:** 10.1590/1984-0462/2023/41/2021312

**Published:** 2023-03-13

**Authors:** Clara dos Santos de Andrades, Victória Volkweis Poletti, Vera Elizabeth Closs, Andreia da Silva Gustavo, Margareth da Silva Oliveira, Márcio Vinícius Fagundes Donadio, Ana Maria Pandolfo Feoli

**Affiliations:** a Pontifícia Universidade Católica do Rio Grande do Sul, Porto Alegre, RS, Brazil.

**Keywords:** Adolescent, Obesity, Cardiovascular diseases, Atherosclerosis, Lipid metabolism disorders, Body mass index, Adolescente, Obesidade, Doenças cardiovasculares, Aterosclerose, Transtornos do metabolismo dos lipídeos, Índice de massa corporal

## Abstract

**Objective:**

To analyze the lipid profile and cardiovascular risk of overweight and obese adolescents and correlate the findings with anthropometric measurements.

**Methods:**

This is a cross-sectional study on overweight and obese adolescents of both sexes (aged 14 to 18 years old). The collected variables were sex, weight, height, age, total cholesterol, triglycerides, High-density lipoprotein (HDL) and low-density lipoprotein (LDL). The Atherogenic Index of Plasma and Castelli Risk Indices I and II were calculated. These indices were classified into cutoff points to stratify cardiovascular risk. The anthropometric profile was evaluated by Z score according to Body Mass Index for age. Significance level was considered as p≤0.05.

**Results:**

A total of 146 adolescents participated in the study; the mean age was 16.4±1.1 years and most of them were girls (74.7%) and obese (52.7%). The prevalent dyslipidemias were high triglycerides (47.9%), LDL (26.7%), total cholesterol (37.7%), and low HDL (46.6%). Most adolescents presented increased atherogenic risk according to the Atherogenic Index of Plasma (55.5%); 15.1% presented high cardiovascular risk according to Castelli Risk Index I; and 13.7%, according to Castelli Risk Index II. Boys presented higher values of anthropometric measurements and Castelli Risk Indices I and II in relation to girls — who, conversely, presented higher values of HDL. There was a positive correlation of the Z score with Atherogenic Index of Plasma and a negative correlation with HDL.

**Conclusions:**

The adolescents of the study presented high prevalence of cardiovascular and atherogenic risk according to the evaluated indices. In addition, the increased cardiovascular risk was correlated with higher Body Mass Index.

## RESUMO

**Objetivo:**

Analisar o perfil lipídico e os índices de risco cardiovascular de adolescentes com sobrepeso e obesidade e correlacionar os achados com medidas antropométricas.

**Métodos:**

Estudo transversal com adolescentes com sobrepeso ou obesidade de ambos os sexos (14 a 18 anos). Foram coletadas as variáveis: sexo, peso, altura, idade, colesterol total, triglicerídeos, lipoproteína de alta densidade (HDL-c) e lipoproteína de baixa densidade (LDL-c). Calcularam-se o índice aterogênico plasmático e os índices de Castelli I e II. Eles foram classificados em pontos de corte para estratificar o risco cardiovascular. O perfil antropométrico foi avaliado por meio do escore Z com base no índice de massa corporal para a idade. Considerou-se o nível de significância p≤0,05.

**Resultados:**

Foram incluídos 146 adolescentes, com média de idade de 16,4±1,1 anos, a maioria do sexo feminino (74,7%) e obesa (52,7%). As dislipidemias prevalentes foram: triglicerídeos (47,9%), LDL-c (26,7%), colesterol total (37,7%) elevado e HDL-c baixo (46,6%). A maioria apresentou risco aterogênico aumentado pelo índice aterôgenico plasmático (55,5%); 15,1% apresentaram alto risco cardiovascular segundo o índice de Castelli I e 13,7%, segundo o índice de Castelli II. Os meninos apresentaram valores superiores de medidas antropométricas e índices de Castelli I e II em relação às meninas, que, por outro lado, apresentaram valores superiores de HDL-c. Houve correlação positiva do escore Z com o índice aterôgenico plasmático e negativa com HDL-c.

**Conclusões:**

Os adolescentes do estudo apresentaram alta prevalência de risco cardiovascular e aterogênico conforme os índices avaliados. Além disso, o risco cardiovascular aumentado foi correlacionado com maior índice de massa corporal.

## INTRODUCTION

Currently, there is a high prevalence of overweight or obese adolescents in Brazil.^1^ Obesity is a multifactorial disease characterized by excess weight and accumulation of body fat, caused by the imbalance between energy consumption and expenditure.^2^ Obesity is a serious public health issue, as it falls within the most important risk factors for cardiovascular diseases (CVDs). According to the Pan American Health Organization (PAHO/WHO), CVDs are the leading cause of death in Brazil.^3^ In addition, their severity extends to the socioeconomic sphere, and the level of expenditures in this context is worrisome. Recently, an analysis of the economic impact in Brazil was published, which found that costs have significantly increased in the last five years. In addition, these costs are estimated to increase more and more as the population of young people with risk factors ages and the prevalence of cardiovascular events increases.^4^

Although the clinical manifestations of CVDs occur only in adulthood, asymptomatic manifestations may still be present in adolescence.^5^ Excess body adiposity is related to the presence of dyslipidemia, identified by increases in the concentration of total cholesterol (TC), triglycerides (TG), and low-density lipoprotein (LDL) and by the decrease in high-density lipoprotein (HDL).^6^

Indices based on lipid markers have been studied to stratify cardiovascular risk (CVR). Castelli Risk Indices I and II (CI-1 and CI-2) prove to be effective in evaluating CVR.^7^ CI-1 is estimated by the ratio between TC and HDL; and CI-2 is calculated by the ratio between LDL and HDL.^8,9^ Another indicator that can be used as a predictor of atherogenic risk is the Atherogenic Index of Plasma (AIP), estimated using the formula log10 (TC/HDL).^10^

The use of indices for this population represents a simple investigation of certain risk factors, as well as a prior diagnosis, which guarantees better management in combating the occurrence of CVD. It should be noted that early detection of cardiovascular risk factors can reduce the chances of future complications and the serious consequences of CVD.

In view of the importance of early evaluating CVR, as well as the lack of studies that use CVR indices in adolescents, the objective of the present study is to analyze the lipid profile and CVR according to CI-1, CI-2, and AIP and to correlate the findings with anthropometric measurements of overweight and obese adolescents.

## METHOD

**Figure 1.**
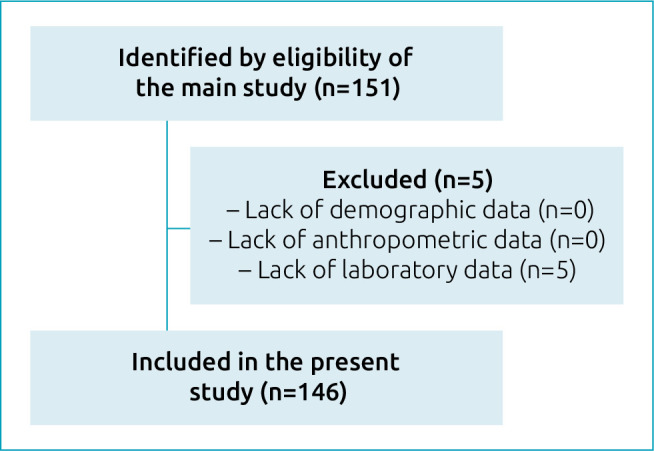
Flowchart of study participants.

This is a cross-sectional study, conducted with secondary data from a larger study entitled *Randomized clinical trial of a motivational interdisciplinary intervention based on the transtheoretical model of change for lifestyle modification in overweight/obese adolescents: MERC study protocol*.^11^ The sample derives from the database of the main study, which included adolescents of both sexes aged between 14 and 18 years and who are overweight or obese (≥Z score +1). The sample was recruited by convenience, by disclosing the research in print media, social networks, television, and the radio. The present study included only participants who had complete data in the original database for the analysis of the studied outcome, as specified in the flowchart (Figure 1).

The study was registered in the Clinical Trial Registry (NCT02455973) and in the Brazilian Registry of Clinical Trials (RBR-234nb5), and it was approved by the Research Ethics Committee of Pontifícia Universidade Católica do Rio Grande do Sul (PUCRS), registered under Certificate of Presentation for Ethical Consideration (CAAE) 36209814.6.0000.5336. All parents and/or legal guardians signed the Informed Consent Form, and the adolescents signed the Assent Form. A declaration of confidentiality of the data was signed by the authors to carry out the present study.

Sociodemographic (age and sex) and anthropometric (weight and height) variables, in addition to biochemical data (serum levels of TC, TG, and HDL), were collected. Blood collection to record biochemical data was performed by trained professionals, through venipuncture after 8 hours of fasting, and blood samples were analyzed at the biochemical laboratory of Hospital São Lucas at PUCRS (HSL). LDL was estimated by the Friedewald equation.^12^ Weight was obtained with the individuals in the orthostatic position, with minimal clothing, without shoes, using a digital scale (G-Tech, Glass 1 FW, Rio de Janeiro, Brazil) with an accuracy of 100 g. Height was collected with the participants barefoot, with their feet in a parallel position and their ankles together. Height measurements were obtained using a portable stadiometer (AlturaExata, TBW, São Paulo, Brazil) with an accuracy of 1 mm. Once these measurements were made, the assessment of anthropometric characteristics was performed based on body mass index for age (BMI-for-age), according to the age group of the sample. For interpretation, the Z score was used: overweight (≥Z score +1 and <Z score +2), obesity (≥Z score +2 and ≤Z score +3), and severe obesity (>Z score +3).^13^

For the survey of cardiovascular risk scores, the following indices were calculated: CI-1, CI-2, and AIP. CI-1 was estimated using the formula TC/HDL, and CI-2 by using the formula LDL/HDL. Cutoff points were adopted for the pediatric population based on the large study conducted by Navarra, with high risk above 3.5 for CI-1 and above 2.2 for CI-2.^14^ AIP was estimated by the formula log10 (TG/HDL). Risk classification according to AIP was divided into three categories: low risk (<0.11); intermediate risk (between ≥0.11 and ≤0.21); and increased risk (>0.21).^15^ For the classification of dyslipidemias, the reference values for adolescents according to Faludi were adopted.^16^

Analyses were performed using the *Statistical Package for the Social Sciences* (SPSS) software, version 21.0 (Inc., Chicago, Illinois, USA). The normality of the distribution of numerical data was verified by the Kolmogorov-Smirnov Test and by analyzing the histogram of these data. Quantitative variables with normal distribution were described by mean and standard deviation, and the nonparametric AIP variable, by median and interquartile range. Pearson’s chi-square and Fischer’s exact tests were used to assess the association between categorical variables, and Student’s *t*-test, Mann-Whitney test, and one-way analysis of variance (ANOVA) were used to compare quantitative measures. The continuous data were correlated with the Pearson’s and Spearman’s correlation tests, according to the normality or not of the data, and classified according to Mukaka.^17^ Analyses were performed considering a 95% confidence interval (CI) (p<0.05).

## RESULTS

The characteristics of the sample were described in Table 1. A total of 146 adolescents were evaluated, with mean age of 16.4±1.1 years, and most of them were girls (74.7%) and obese (52.7%). Boys presented higher Z scores and, more frequently, were severely obese, while girls were more frequently overweight or obese (Table 1).

**Table 1.** Sociodemographic, anthropometric, and clinical characteristics of the total sample, according to sex of overweight and obese adolescents (n=146)

Characteristics	Total sample	Sex		p-value
		Boys n=37	Girls n=109	
**Sociodemographic**				
Age (years)	16.4±1.1	16.1±1.2	16.5±1.1	0.085*
**Anthropometric**				
Z score	2.9±0.9	3.4±0.9	2.8±0.8	<0.001*
Classification				
Overweight	14.0 (9.6)	1.0 (2.7)	13.0 (11.9)	0.013^†^
Obesity	77.0 (52.7)	15.0 (40.5)	62.0 (56.9)	
Severe obesity	55.0 (37.7)	21.0 (56.8)	34.0 (31.2)	
**Clinical**				
Triglycerides (mg/dL)	95.3±45.0	86.2±30.9	98.4±48.6	0.079*
Classification				
Adequate	76.0 (52.1)	21.0 (56.8)	55.0 (50.5)	0.508^‡^
Altered	70.0 (47.9)	16.0 (43.2)	54.0 (49.5)	
HDL (mg/dL)	48.8±13.7	41.8±8.5	51.1±14.4	<0.001*
Classification				
Adequate	78.0 (53.4)	11.0 (29.7)	67.0 (61.5)	0.001^‡^
Altered	68.0 (46.6)	26.0 (70.3)	42.0 (38.5)	
LDL (mg/dL)	96.0±27.8	96.9±28.8	95.7±27.6	0.825*
Classification				
Adequate	107.0 (73.3)	26.0 (70.3)	81.0 (74.3)	0.631^‡^
Altered	39.0 (26.7)	11.0 (29.7)	28.0 (25.7)	
Total cholesterol (mg/dL)	163.9±33.3	155.9±32.4	166.6±33.3	0.093*
Classification				
Adequate	91.0 (62.3)	26.0 (70.3)	65.0 (59.6)	0.249^‡^
Altered	55.0 (37.7)	11.0 (29.7)	44.0 (40.4)	

BMI: body mass index; HDL: high-density lipoprotein; LDL: low-density lipoprotein. Quantitative variables with normal distribution are presented by mean±standard deviation; nonparametric variables, by median and interquartile range; and categorical variables, by absolute and relative values. *Student’s *t*-test; ^†^Fischer’s exact test; ^‡^Pearson’s chi-square test.

When comparing the lipid profile, girls presented higher values than boys for HDL, with a higher percentage of adequacy, and lower values for CI-1 and CI-2. The prevalence of dyslipidemia among the investigated participants was 47.9% (95%CI 36.2 and 53.9%) for high TG values; 26.7% (95%CI 12.8 and 33.8%) for LDL; and 37.7% (95%CI 24.9 and 44.2%) for TC. As for HDL, 46.6% (95%CI 34.7 and 52.6%) of the adolescents presented values below those recommended. Regarding the indices, most adolescents presented increased atherogenic risk according to AIP (55.5%; 95%CI 44.7 and 61.0%). Regarding the Castelli Risk Indices, 15.1% (95%CI 0.1 and 22.7%) and 13.7% (95%CI -1.4 and 21.4%) presented high cardiovascular risk according to CI-1 and CI-2, respectively (Tables 1 and 2).

**Table 2.** Classification of cardiovascular risk parameters of the total sample, according to sex of overweight and obese adolescents (n=146).

Risk parameters	Total sample	Sex		p-value
		Boys n=37	Girls n=109	
Castelli Risk Index 1	3.5±1.0	3.8±1.0	3.4±1.0	0.048*
Classification				
No cardiovascular risk	124.0 (84.9)	34.0 (91.9)	90.0 (82.6)	0.171^†^
High cardiovascular risk	22.0 (15.1)	3.0 (8.1)	19.0 (17.4)	
Castelli Risk Index 2	2.1±0.9	2.4±0.8	2.0±0.9	0.026*
Classification				
No cardiovascular risk	126.0 (86.3)	32.0 (86.5)	94.0 (86.2)	0.970^†^
High cardiovascular risk	20.0 (13.7)	5.0 (13.5)	15.0 (13.8)	
Atherogenic Index of Plasma	0.2 (0.1-0.4)	0.3 (0.1-0.4)	0.2 (0.1-0.4)	0.162^‡^
Classification				
Low risk	36.0 (24.7)	6.0 (16.2)	30.0 (27.5)	0.216^†^
Intermediate risk	29.0 (19.9)	6.0 (16.2)	23.0 (21.1)	
Increased risk	81.0 (55.5)	25.0 (67.6)	56.0 (51.4)	

*Student’s *t*-test; ^†^Pearson’s chi-square test; ^‡^Mann-Whitney test.

The results of the correlation analyses between the Z score and the cardiovascular risk parameters are described in Table 2. In the total sample, the Z score showed a positive correlation with AIP, and a negative correlation with HDL, significantly. There was a positive correlation with the Castelli Risk Indices I and II, although negligible. Among boys, the Z score was positively correlated with AIP and TG.

The results of the comparison between the Z score and the other cardiovascular risk classifications, according to the indices, are presented in Tables 3 and 4. In the total sample, the Z score was higher in individuals with low HDL and increased risk according to AIP. The Z score was lower in individuals with high TC. Among boys, the Z score was higher in those with increased risk according to AIP. Conversely, among girls, it was higher in those with low HDL.

**Table 3.** Correlation between Z score and cardiovascular risk parameters of overweight and obese adolescents (n=146).

Clinical variables of cardiovascular risk	Z score					
	Total sample		Sex			
			Boys n=37		Girls n=109	
	*r*	p-value	*r*	p-value	*r*	p-value
Triglycerides (mg/dL)	0.094	0.259	0.334	0.043	0.096	0.320
HDL (mg/dL)	−0.381	<0.001	−0.317	0.056	−0.331	<0.001
LDL (mg/dL)	−0.001	0.987	0.010	0.953	−0.014	0.887
Total cholesterol (mg/dL)	−0.133	0.109	−0.011	0.950	−0.126	0.191
Castelli Risk Index 1	0.281	0.001	0.257	0.125	0.244	0.011
Castelli Risk Index 2	0.250	0.002	0.203	0.227	0.209	0.029
	***s***	**p**	***s***	**p**	***s***	**p**
Atherogenic Index of Plasma	0.302	<0.001	0.451	0.005	0.211	0.028

*r*: Pearson’s correlation; *s*: Spearman’s correlation. HDL: high-density lipoprotein; LDL: low-density lipoprotein.

**Table 4.** Comparison between Z score means, according to the classification of cardiovascular risk parameters of overweight and obese adolescents (n=146).

Cardiovascular Risk Parameters (Classification)	Z score								
	Total sample			Sex					
				Boys n=37			Girls n=109		
	n	Mean±SD	p-value	n	Mean±SD	p-value	n	Mean±SD	p-value
Triglycerides									
Adequate	76.0	2.9±0.8	0.287	21.0	3.2±0.9	0.095	55.0	2.8±0.8	0.599
Altered	70.0	3.0±0.9		16.0	3.7±0.8		54.0	2.8±0.8	
HDL									
Adequate	78.0	2.7±0.7	0.002	11.0	3.2±0.7	0.364	67.0	2.7±0.7	0.040
Altered	68.0	3.2±0.9		26.0	3.5±0.9		42.0	3.0±0.9	
LDL									
Adequate	107.0	3.0±0.9	0.347	26.0	3.4±0.9	0.610	81.0	2.8±0.8	0.310
Altered	39.0	2.8±0.9		11.0	3.3±0.8		28.0	2.7±0.8	
Total cholesterol									
Adequate	91.0	3.1±0.9	0.022	26.0	3.5±0.9	0.296	65.0	2.9±0.8	0.084
Altered	55.0	2.7±0.8		11.0	3.2±0.8		44.0	2.6±0.8	
Castelli Risk Index 1									
No risk	124.0	2.9±0.9	0.264	34.0	3.4±0.8	0.721	90.0	2.7±0.8	0.052
High risk	22.0	3.1±0.9		3.0	3.2±1.4		19.0	3.1±0.8	
Castelli Risk Index 2									
No risk	126.0	2.9±0.8	0.213	32.0	3.4±0.9	0.757	94.0	2.7±0.8	0.081
High risk	20.0	3.2±0.9		5.0	3.3±1.0		15.0	3.1±0.9	
Atherogenic Index of Plasma									
Low risk	36.0	2.7^a^±0.7		6.0	2.6^a^±0.4		30.0	2.7±0.7	
Intermediate risk	29.0	2.7±0.9	0.011	6.0	3.2±1.1	0.033	23.0	2.6±0.8	0.236
Increased risk	81.0	3.1^b^±0.9		25.0	3.6^b^±0.8	56.0	2.9±0.9		

HDL: high-density lipoprotein; LDL: low-density lipoprotein.

## DISCUSSION

The results of the present study demonstrated that the prevalence of cardiovascular and atherogenic risk among adolescents was high. Furthermore, it was possible to observe, with the use of the indices to stratify the risk, that the risk is high according to the BMI classification, even in a sample exclusively composed of overweight and obese individuals. This statement can be evidenced by the results of correlation between the anthropometric characteristic and the indices’ ratings.

Nationwide data, represented by the *Study of Cardiovascular Risk in Adolescents* (ERICA), which evaluated 38,069 adolescents, showed that a significant proportion of Brazilian adolescents present changes in plasma lipids. With emphasis on the south region, in the same study, low HDL (36.9%) and high TC (22.8%), TG (8.2%), and LDL (3.5%) were reported.^5^

The present study demonstrated a significantly higher percentage of prevalence of lipid alterations compared with the ERICA findings. Even considering the differences in the samples, such as, mainly, the age interval and the number of participants, it is worth noting the fact that, in this study, the participants are exclusively overweight or obese adolescents. This reinforces the possibility that overweight and obesity are important factors of lipid changes in adolescence.

Research shows that the main dyslipidemia associated with obesity is characterized by increases in TG and decreased HDL.^18^ It is noteworthy that low HDL and hypertriglyceridemia were the most prevalent dyslipidemias in this study, which is in line with this statement. HDL is an important protective factor against the development of atherosclerosis.^16^ It has already been observed, in practice, that patients with atherosclerotic coronary heart disease did not have high LDL, but had low HDL.^19^ Hence, the application of indices that relate plasma lipid levels becomes relevant to estimate CVR, rather than their isolated assessment.

Regarding the assessed indices, the results indicate a significant proportion of adolescents with CVR. Most of them had an increased atherogenic risk, according to AIP. In addition, 15.1 and 13.7% presented high CVR according to CI-1 and CI-2, respectively. Concerning atherogenic risk, a study that evaluated atherogenic indices in a population of Spanish adolescents found that AIP was strongly associated with the prognosis of metabolic syndrome.^20^

Another investigation conducted in northeastern Brazil, on 448 adolescents, correlated AIP with CI-1 and CI-2 and other risk predictors such as dyslipidemia, physical inactivity, overweight, and obesity. The authors found a prevalence of 36.2 and 31% of high risk according to CI-1 and CI-2, respectively. In addition, the results of this study showed a positive correlation of AIP with most of the evaluated CVR predictors. These data suggest that the indices prove to be efficient as markers of cardiovascular risk in overweight adolescents.^21^

Accordingly, the findings of the present study demonstrated that higher AIP values were significantly associated with overweight and obesity and low HDL. In other words, these data also indicate a worrisome association between the nutritional profile of the studied population and the presence of dyslipidemia and CVR.

A cross-sectional study conducted on 807 Brazilian adolescents evaluated the prevalence of dyslipidemia and CVR according to CI-1 and CI-2. The results showed that the indices were more prevalent markers in adolescents who were overweight, in both sexes and at all ages. Thus, they emphasize that CI-1 and CI-2 are effective predictors of CVR in adolescents who are overweight. Therefore, the outcome of the study is in line with the present results.^22^ In southern Brazil, Reuter et al. also found a significant association between dyslipidemia and obesity in the 1,254 adolescents evaluated.^23^

We can state that obesity is among the most prevalent association factors of dyslipidemia in the young population. It is worth mentioning that dyslipidemia is responsible for the incidence of atherosclerosis, which can begin in childhood and gradually progress into adulthood.^20^ The Brazilian Society of Cardiology emphasizes that the population is at risk of a premature CVD epidemic in the future, due to the increase in risk factors in the pediatric age group.^16^

It should be noted that the main limitation of the study is the limited number of adolescents included. Nonetheless, more research is needed, which includes larger samples and, especially, other regions of Brazil, seeking to reinforce the findings of this study.

Lipid changes are an important health issue among young people, and CVD prevention should be focused on this stage.^24^ Therefore, measures that modify risk factors to reduce or avoid future complications should be implemented in this specific population. For this purpose, early screening of CVR by using scores allows a better understanding of the pathogenesis and how to appropriately intervene.

The results of our study pointed to the high prevalence of CVR and atherogenic risk according to the indices in the studied population. In addition, increased risk was correlated with higher BMI.
